# Microorganisms Move a Short Distance into an Almond Orchard from an Adjacent Upwind Poultry Operation

**DOI:** 10.1128/AEM.00573-20

**Published:** 2020-07-20

**Authors:** Christopher G. Theofel, Thomas R. Williams, Eduardo Gutierrez, Gordon R. Davidson, Michele Jay-Russell, Linda J. Harris

**Affiliations:** a Department of Food Science and Technology, University of California, Davis, Davis, California, USA; b Western Center for Food Safety, University of California, Davis, Davis, California, USA; Rutgers, The State University of New Jersey

**Keywords:** almond, bioaerosol, dust, microbiome, orchard, phyllosphere, poultry

## Abstract

Over a 2-year period, drag swabs of orchard soil surface and air, soil, and almond leaf samples were collected in an almond orchard adjacent to (35 m from the first row of trees) and downwind from a poultry operation and in two almond orchards (controls) that were surrounded by other orchards. Samples were evaluated for aerobic plate count, generic Escherichia coli, other coliforms, the presence of *Salmonella*, bacterial community structure (analyzed through sequencing of the 16S rRNA gene), and amounts of dry solids (dust) on leaf surfaces on trees 0, 60, and 120 m into each orchard. E. coli was isolated from 41 of 206 (20%) and 1 of 207 (0.48%) air samples in the almond-poultry and control orchards, respectively. *Salmonella* was not isolated from any of the 529 samples evaluated. On average, the amount of dry solids on leaves collected from trees closest to the poultry operation was more than 2-fold greater than from trees 120 m into the orchard or from any of the trees in the control orchards. Members of the family *Staphylococcaceae*—often associated with poultry—were, on average, significantly (*P < *0.001) more abundant in the phyllosphere of trees closest to the poultry operation (10% of relative abundance) than in trees 120 m into the orchard (1.7% relative abundance) or from any of the trees in control orchards (0.41% relative abundance). Poultry-associated microorganisms from a commercial operation transferred a short distance into an adjacent downwind almond orchard.

**IMPORTANCE** The movement of microorganisms, including foodborne pathogens, from animal operations into adjacent plant crop-growing environments is not well characterized. This study provides evidence that dust and bioaerosols moved from a commercial poultry operation a short distance downwind into an almond orchard and altered the microbiome recovered from the leaves. These data provide growers with information they can use to assess food safety risks on their property.

## Abstract (precis)

The movement of microorganisms, including foodborne pathogens, from animal operations into adjacent plant crop-growing environments is not well characterized. This study provides evidence that dust and bioaerosols moved from a commercial poultry operation a short distance downwind into an almond orchard and altered the microbiome recovered from the leaves. These data provide growers with information they can use to assess food safety risks on their property.

## INTRODUCTION

California is home to a robust and diverse agriculture sector that includes more than 70,000 farms on more than 10 million hectares (1). In 2017, California had an average annual inventory of nearly 7 million head of beef and dairy cattle and 13 million laying hens and accounted for 46% and 57% of the U.S. fruit and nut production and vegetable production, respectively. In addition, California accounts for >99% of the almond, pistachio, and walnut production in the United States. Slightly over 1 billion kg of almond kernels was produced in 2017 on approximately 400,000 bearing hectares (1). An unintended result of the breadth and scope of California agriculture is that fruits, nuts, and vegetables can be grown in close proximity to livestock operations.

The risk of pathogen transport from livestock operations to neighboring food crops is not well characterized. Epidemiological evidence has linked cattle to outbreaks of foodborne illness associated with leafy greens grown nearby, with irrigation or other agricultural water uses or wildlife potentially serving as an intermediary (2–6). Birds and insects have the potential to move pathogens from animal operations into growing environments (7–12). Bioaerosols capable of carrying pathogenic bacteria have been described to occur in cattle feedlots, in poultry and swine houses, and during field application of manure (13–18). Airborne generic Escherichia coli was found both inside and immediately outside poultry houses; levels of E. coli in the air were linked to the levels found in the litter (19). E. coli O157:H7 could be recovered from 3.5%, 2.2%, and 1.8% of leafy green samples collected over a 4-month period at 60, 120, and 180 m, respectively, from a cattle feedlot; airborne transport of the pathogen was implicated, and pen dryness and animal activities that generate airborne dust were the noted risk factors (13). Transfer of pathogenic E. coli from livestock to vegetable production systems may be influenced by both the size of the animal operation and proximity to the farm (8).

There are few studies examining the risks associated with livestock operations in close proximity to tree crops. E. coli (on apples or kiwi fruit) and Staphylococcus aureus (on kiwi fruit) were isolated from orchard fruit collected preharvest above the soil, or from irrigation or other surface water, suggesting the possibility of a bioaerosol vehicle (20–22). However, data evaluating movement of microorganisms from livestock operations via bioaerosols into tree crops are lacking (16).

The Produce Safety Rule (23), part of the U.S. Food Safety Modernization Act, requires an assessment of areas in which covered (included) produce crops are grown, harvested, packed, or held for evidence of potential contamination, such as animal intrusion, animal excreta, or crop destruction. Additional details related to prevention of cattle waste runoff into produce fields are included in the rule’s Draft Guidance for Industry (24). In contrast, the California Leafy Green Products Handler Marketing Agreement (CA LGMA) (25) and Arizona Leafy Greens Marketing Agreement (AZ LGMA) (26) require an assessment of the risk of crop contamination through bioaerosols. Current CA LGMA and AZ LGMA guidance documents provide suggested distances from concentrated animal feeding operations to leafy green plantings that are based on the size and type of animal operation. The guidelines acknowledge that the risk of pathogen transport from livestock operations to surrounding food crops is not well characterized.

It is not known how many California almond orchards are adjacent to livestock operations or whether this proximity influences contamination of the almond crop. The aim of the present study was to examine the microbiota of almond orchards (both in close proximity to and at a distance from a poultry operation) and to evaluate the potential for transfer of microorganisms from a poultry operation adjacent to and downwind of an almond orchard. We hypothesized that the microbiota found in a commercial almond orchard—in the air, soil, and tree phyllosphere—is impacted by organisms originating from nearby poultry operations.

## RESULTS

### Weather conditions.

The maximum wind speed recorded during any orchard visit at any location was 2.6 m/s. When averaged over all orchard locations, wind speed, relative humidity, and temperature ranged from 0.2 to 1.2 m/s, 40.8 to 75.8%, and 15.3 to 25.8°C, respectively. A complete summary of recorded meteorological data by orchard visit is presented in Table S1 in the supplemental material.

### Isolation and enumeration of bacteria from orchard air.

Totals of 206, 171, and 36 air samples were collected in orchards AP (2013 and 2014), AC1 (2013 and 2014), and AC2 (2014 only), respectively. Aerobic plate counts in the air samples ranged from 1.1 to >3.4 (the upper limit of detection [ULOD]) log CFU/m^3^ of air. An outcome of running the air samplers at maximum setting was that aerobic plate counts often exceeded the ULOD. In general, the counts increased through the calendar year. In June 2013, counts in AP ranged from 2.3 to 2.9 log CFU/m^3^, and counts for all samples in AC1 were above the ULOD (see Tables S2 and S3 in the supplemental material). In both orchards at mid-harvest, all but one air sample had counts above the ULOD. In 2014, aerobic plate counts were determined in June and July: for June, counts were above the ULOD for all six samples in AP and AC1 and ranged from 2.6 to 3.3 in AC2; for July, counts were above the ULOD, with the exception of three samples in AC1 and one sample in AC2. The counts were consistent within orchards on a sampling day; greater differences were seen among sampling dates.

Coliforms other than E. coli were isolated from at least one air sample from each orchard at all but one collection time: AP (69%; 143/206), AC1 (80%; 136/171), and AC2 (19%; 7/36) (Table 1). E. coli was detected from at least one AP air sample for all but one collection time (20% of all samples; 41/206), never isolated from AC1 (0%; 0/171), and isolated from a single air sample from AC2 (3%; 1/36) (Table 1). Air samples were enriched for *Salmonella* in 2013 only; no positives were detected among the 324 samples evaluated (0/189 for AP and 0/135 for AC1).

**TABLE 1:** Proportion of air and soil enrichment samples positive for E. coli and other coliforms in almond orchards in 2013 and 2014^*a*^

Orchard sampling period	Total no. of positives/total no. of samples											
	Air						Soil					
	Other coliforms			E. coli			Other coliforms			E. coli		
	AP	AC1	AC2	AP	AC1	AC2	AP	AC1	AC2	AP	AC1	AC2
2013												
March	9/27	ND^*b*^	ND	2/27	ND	ND	ND	ND	ND	ND	ND	ND
April	13/27	ND	ND	9/27	ND	ND	ND	ND	ND	ND	ND	ND
May	23/27	23/27	ND	0/27	0/27	ND	ND	ND	ND	ND	ND	ND
June	25/27	26/27	ND	8/27 A	0/27 B	ND	11/11	9/9	ND	3/11	0/9	ND
July	12/18	23/27	ND	1/18	0/27	ND	11/11	9/9	ND	2/11	2/9	ND
August	10/18	18/27	ND	10/18 A	0/27 B	ND	ND	ND	ND	ND	ND	ND
Mid-harvest	27/27	26/27	ND	4/27	0/27	ND	11/11	9/9	ND	11/11 A	5/9 B	ND
2014												
June	12/18 A	9/18 A	0/18 B	3/18	0/18	1/18	9/9	9/9	9/9	1/9	0/9	2/9
July	12/17	11/18	7/18	4/17 A	0/18 B	0/18 B	9/9	9/9	9/9	2/9	1/9	1/9
Total	143/206 B	136/171 A	7/36 C	41/206 A	0/171 B	1/36 B	51/51	45/45	18/18	19/51 A	8/45 B	3/18 B

a Orchard designations: AP, Almond Poultry; AC1, Almond Control 1; AC2, Almond Control 2. Within rows for orchard sample type and organism, proportions with different uppercase letters are significantly different (*P *< 0.05).

b ND, not determined (AC1 was not sampled until May 2013, and AC2 was not sampled until 2014).

### Dry solid (dust) assessment in the orchard phyllosphere.

**FIG 1:**
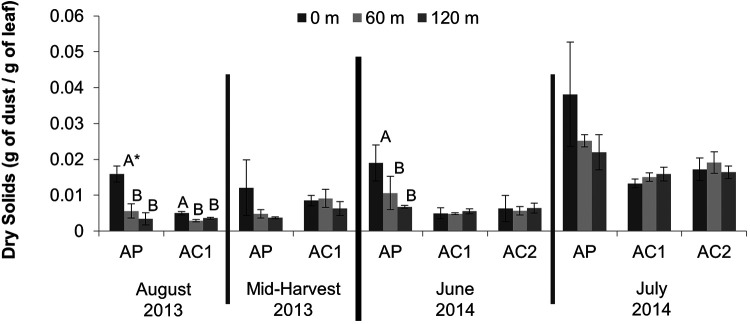
Average levels of dry solids (gram of dust per gram of leaf) as determined by dry weight of dust rinsed off orchard leaves (25.0 ± 0.2 g) collected from rows at 0, 60, or 120 m from the edge of the orchard closest to the poultry operation or entry road (*n *≥ 3). *, different letters indicate significant differences (*P < *0.05) in dust levels between rows within an orchard.

The dry solids on the leaf surfaces in orchards AP, AC1, and AC2 were quantified in 2013 (August and mid-harvest) and 2014 (June and July) (Fig. 1). In AP, the level of dry solids was significantly higher at 0 m than at 60 or 120 m in August 2013 (*P = *0.0019) and June 2014 (*P = *0.0231). In August 2013, averages of 0.0159, 0.0056, and 0.0034 g of dust/g of leaf were recovered from AP rows at 0, 60, and 120 m, respectively. In June 2014, averages of 0.0189, 0.0106, and 0.00676 g of dry solids/g of leaf were recovered from AP rows at 0, 60, and 120 m, respectively. At other sampling times, higher but not significant differences in dust levels were found in AP at 0 m than at 60 or 120 m.

In all cases except for mid-harvest 2013, the level of dust recovered from AP at 0 m was significantly greater (August 2013, *P = *0.0022; June 2014, *P = *0.0012; and July 2014, *P = *0.0022) than the levels recovered for any of the rows from AC1 or AC2. For AC1, significant (*P < *0.0001) differences in dry solids between 0 m and the other rows occurred in August 2013, with averages of 0.0049, 0.0029, and 0.0036 g of dry solids/g of leaf for the rows at 0, 60, and 120 m, respectively. No significant differences were observed among the rows on any other sampling date for either of the control orchards.

### Detection of E. coli, other coliforms, and *Salmonella* from the orchard floor.

Topsoil samples were collected from orchards AP and AC1 in 2013 and 2014 and from orchard AC2 in 2014. Coliforms other than E. coli were detected in every soil sample collected from all orchards: AP (51/51), AC1 (45/45), and AC2 (18/18) (Table 1). E. coli was detected in soil from at least one location on every visit (5/5) for AP and on 5 of 7 visits for the control orchards. Excluding the mid-harvest sampling, E. coli was detected in 15% (14/94) of all orchard soil samples. During mid-harvest, 100% (11/11) and 56% (5/9) of soil samples were positive for AP and AC1, respectively. AC2 was not visited during mid-harvest.

In 2013 and 2014, totals of 45, 21, and 6 pooled orchard surface drag swabs were collected from AP, AC1, and AC2, respectively. *Salmonella* was not isolated from any of the pooled drag swabs. There were no *Salmonella*-positive enrichments among the 133 soil samples analyzed in 2013 (0/88 for AP and 0/45 for AC1); soil samples were not tested for *Salmonella* in 2014.

### Bacterial community structures within an almond orchard.

The bacterial communities associated with the almond orchard phyllosphere, soil, and air were investigated by 16S rRNA gene sequencing. Using a 97% similarity cutoff, 33,900 operational taxonomic units (OTU) were obtained from slightly over 14.5 million high-quality sequences. Sequences per sample ranged from 1,262 to 380,121, with a median of 37,807. Principal-coordinate analysis using the weighted UniFrac community distance metric showed that the bacterial communities associated with each sample type (phyllosphere, soil, and air) were distinct (see Fig. S1 in the supplemental material). The alpha-diversities, as measured by the metrics Chao1 and Shannon, were significantly different (*P < *0.0001 for both metrics) among the three sample types, with soil having the most diversity and air samples the least (see Fig. S2 in the supplemental material).

### Bacterial community changes as influenced by poultry operations.

**FIG 2:**
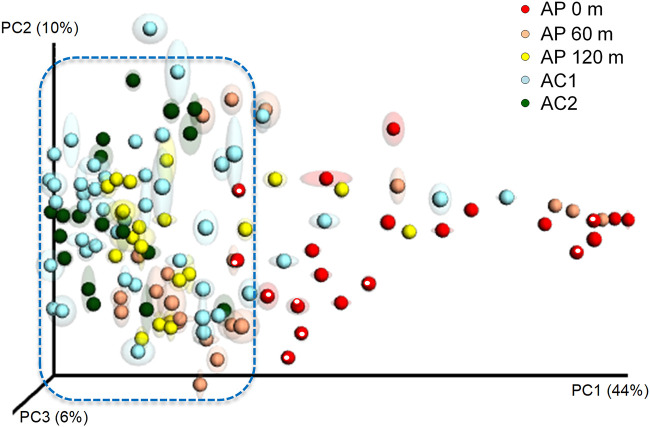
Principal-coordinate analysis of almond orchard phyllosphere samples using the weighted UniFrac community distance metric. White dots within red spheres indicate AP 0-m samples from mid-harvest 2013. AP 0 m, AP 60 m, and AP 120 m, *n *= 21; AC1, *n *= 44; and AC2, *n *= 18.

To assess whether the poultry operation had an impact on orchard community structures, the phyllosphere, air, and soil samples were evaluated. Analysis of similarities (ANOSIM) on weighted and unweighted UniFrac data showed significant differences among the microbiota of the three orchards for both the phyllosphere (weighted, *P = *0.002; unweighted, *P* = 0.033) and soil samples (weighted, *P = *0.001; unweighted, *P = *0.001). Principal-coordinate analysis of weighted UniFrac data further indicated that the microbiota identified among the orchards were distinct for the phyllosphere (Fig. 2) and, to a lesser extent, the soil (see Fig. S3 in the supplemental material). Most of the phyllosphere samples (75%) clustered together, including all of the AC2 samples. Phyllosphere samples from AP had the greatest community dissimilarity among like samples; most (19/21) AP 0-m samples landed outside the main cluster (Fig. 2). The two AP 0-m samples that were in the main cluster and most AP 0-m samples that were closer to the main cluster were from mid-harvest 2013. The microbial communities for three of 21 AP 60-m samples grouped with the most distant AP 0-m samples, but most (16/21) were within the main cluster. The microbiome of roughly 14% relative abundance of both AP 120-m (3/21) and AC1 (6/44) samples was also outside the main cluster.

**FIG 3:**
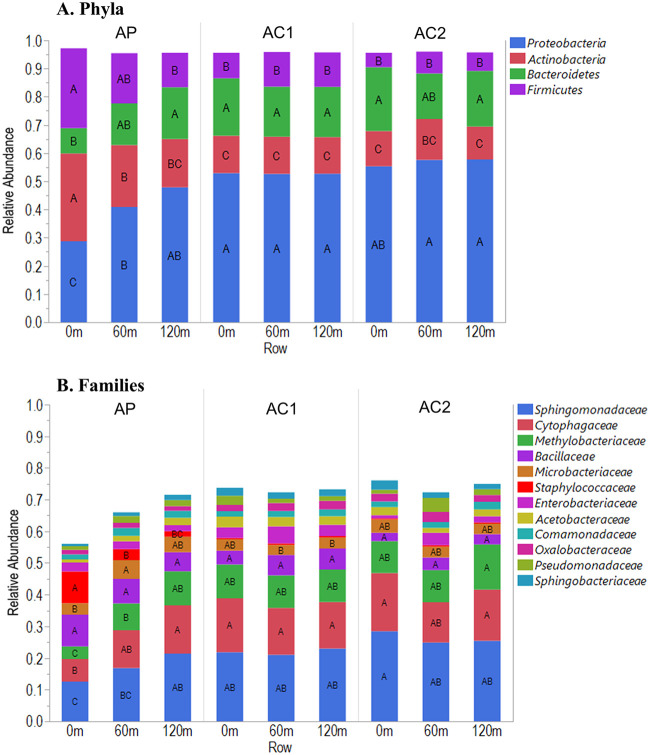
Relative abundances of the most dominant bacterial phyla (A) and families (B) in orchard phyllosphere samples. AP, *n *= 63; AC1, *n *= 45; AC2, *n *= 18. Within a phylum or family, different letters indicate significant differences in relative abundance among all rows. Statistical analysis is shown for the most dominant phyla and families.

For the phyllosphere, these distinct populations can be at least partially explained by significant differences in the relative abundance of the dominant taxonomic groups (Fig. 3). When all phyllosphere samples were considered together, *Proteobacteria*, *Actinobacteria*, *Bacteroidetes*, and *Firmicutes* accounted for more than 95% of the population at the phylum level. For each of these dominant phyla, AP at 0 m had significantly more *Firmicutes* (*P < *0.0001) and *Actinobacteria* (*P < *0.0001) or fewer *Proteobacteria* (*P < *0.0001) and *Bacteroidetes* (*P < *0.0001) than all other orchard rows, with two exceptions: AP at 60 m, which statistically grouped with both AP at 0 m and the other orchard rows for *Firmicutes* and *Bacteroidetes*, and AC2 at 60 m in the case of *Bacteroidetes* (Fig. 3). The same trend was evident for the dominant three bacterial families—*Sphingomonadaceae*, *Cytophagaceae*, and *Methylobacteriaceae*—which made up nearly half (43%) of the phyllosphere population for all samples. The relative abundance of each of the families was significantly lower (*Sphingomonadaceae*, *P < *0.0001; *Cytophagaceae*, *P < *0.0001; *Methylobacteriaceae*, *P < *0.0001) in AP at 0 m than all other rows in AP, AC1, and AC2, with the exception of AP at 60 m, which grouped with AP at 0 m for *Sphingomonadaceae* and *Cytophagaceae* (Fig. 3).

**FIG 4:**
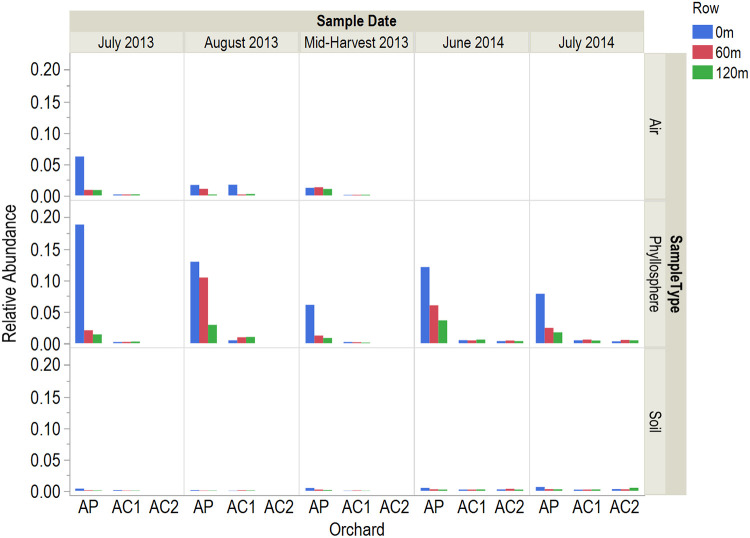
Proportions of bacterial family *Staphylococcaceae* sequences in air, phyllosphere, and soil samples. Air samples were not collected in 2014. AC2 was not sampled until 2014. (Air samples) AP, *n *= 27; AC1, *n *= 24. (Phyllosphere samples) AP, *n *= 63; AC1, *n *= 45; AC2, *n *= 18. (Soil samples) AP, *n *= 51; AC1, *n *= 45; AC2, *n *= 18.

The family *Staphylococcaceae* made up 10% of the phyllosphere population at AP 0 m, a significantly greater amount (*P < *0.0001) than in all other orchard rows, and was the second most abundant family for that row after *Sphingomonadaceae* (with 12%) (Fig. 3). *Staphylococcaceae* made up 3.5% of the AP 60-m and 1.7% of the AP 120-m phyllosphere populations, whereas it made up less than 0.50% of the population in all the control orchard rows (Fig. 3 and 4).

Although there were significant differences between AP at 0 m and the other orchard rows at the phylum and family levels for the orchard soil and air, for the most part the dominant taxa grouped together statistically (see Fig. S4 and S5 in the supplemental material). *Proteobacteria* and *Actinobacteria* dominated, accounting for 54% of the soil community at the phylum level. AP at 0 m had the largest population of *Actinobacteria* (29%), but relative abundance was statistically grouped with all rows except AC1 at 0 and 60 m. At the family level, the bacterial communities found in the soil samples were diverse, with the top 12 families accounting for less than 50% of the community members for all rows. As anticipated, the bacterial communities found in air samples were not diverse in composition, likely, in part, because the enrichment step before sequencing was chosen to favor recovery of members of the *Enterobacteriaceae* family. *Proteobacteria* and *Firmicutes* dominated (99% of the sequences) at the phylum level, whereas *Enterobacteriaceae*, *Bacillaceae*, *Planococcaceae*, and *Pseudomonadaceae* dominated (92% of the sequences) at the family level. A summary of the relative proportions and significant differences between taxonomic groups in the orchard sample types can be found in Tables S4 to S6.

## DISCUSSION

Produce fields and orchards (e.g., vegetables, fruits, and nuts) are sometimes located in close proximity to commercial animal operations. Because animals may shed enteric zoonotic organisms in their feces, good agricultural practices often include grower assessments of potential animal-derived contamination in areas where their crop is grown and harvested (25–27). The movement of dusts and bioaerosols containing microorganisms from animal operations to nearby plant crops represents one possible transfer route; however, this mechanism of transfer is not well characterized (8, 16, 28), making it difficult for growers to appropriately assess their risk. The current study provides evidence that microorganisms moved a short distance into an almond orchard from a poultry operation that was upwind and 35 m from end of the barns to the first row of trees based on (i) the higher prevalence of E. coli in AP air and soil, (ii) the higher levels of dust found in AP at 0 m into the orchard, and (iii) altered phyllosphere microbiota (higher prevalence of the bacterial family *Staphylococcaceae*) found in the phyllosphere in AP at 0 and 60 m into the orchard.

E. coli is sometimes used as an imperfect indicator of fecal contamination. E. coli was isolated from 20% and 37% of AP air and soil samples, respectively; there was no significant difference in the likelihood of finding E. coli in AP air or soil at 0, 60, or 120 m. E. coli was isolated at a significantly higher rate from AP air than air from either control orchard (for AC1, *P < *0.0001; for AC2, *P = *0.0123). In contrast, E. coli was isolated a single time from AC2 and never isolated from AC1. E. coli was isolated in significantly larger amounts in soil from AP than from AC1 (*P = *0.0342) but not AC2 (*P = *0.1071), possibly due to the small number of samples (45 for AC1 versus 18 for AC2) and a lack of statistical power (Table 1).

Almond orchards are typically planted with two or three varieties in separate rows that are harvested at separate times. Mid-harvest samples were taken after the first typical harvest cycle, in which the earliest variety of almond trees were mechanically shaken and the fruits dried on the orchard floor, followed by sweeping into windrows, pickup of fruit, and subsequent removal of debris with application of high-pressure air. Each of these steps involves equipment moving through the orchard and the generation of significant amounts of aerosolized dust (29). Significantly larger amounts of E. coli organisms were isolated in soil collected during the single mid-harvest sampling than from all other samplings for both AP (*P < *0.0001) and AC1 (*P = *0.0109) (AC2 was not sampled at this time). The higher prevalence of E. coli in the soil samples from mid-harvest provides some evidence that harvest activities can spread bacteria throughout an orchard.

Consumption of raw almonds grown in California or Australia has been associated with four documented outbreaks of salmonellosis in 2001, 2004, 2006, and 2012 (30). *Salmonella* can be isolated from raw unprocessed almond kernels at a prevalence of about 1% of 100-g samples (based on almost 15,000 samples collected over a 9-year period) (31–33). The most likely route of contamination is thought to be dusts generated during mechanical pickup of the crop from the orchard floor and subsequent mixing of the kernels, hulls, and shells during postharvest hulling and shelling (34, 35). The best-studied almond outbreak occurred in 2001; the unique *Salmonella* strain linked to this outbreak (serovar Enteritidis phage type 30) was traced back to almond orchards and subsequently isolated from those orchards (from drag swab samples of orchard floor) over a period of 6 years (35, 36).

In the current study, *Salmonella* was never detected from any of the 529 air, soil, and drag swab samples. Access to the poultry operation to test manure and litter samples was not granted, and thus, it was not possible to determine if *Salmonella* was associated with these birds. Commercial laying operations fall under the U.S. Food and Drug Administration’s “Egg Rule” (37) and, in California, the California Shell Egg Food Safety Regulation (38). Accordingly, flocks with more than 3,000 laying hens should be monitored for Salmonella Enteritidis at the receipt of chicks and at the end of production. However, these tests apply only to a single serovar of *Salmonella* (Enteritidis) and would not exclude flocks that were positive for other serovars. The poultry operation next to AP housed an estimated 60,000 layers, meaning that the operation would fall under the rule. There are many other potential sources of *Salmonella* in a production environment, including feral animals and wildlife (5).

**FIG 5:**
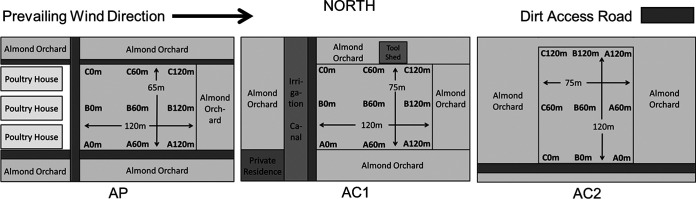
Schematics of collaborating almond orchards.

There was a visible difference in the amount of dust on the leaves in trees in AP at 0 m than in other orchard rows; on average, the level of dry solids on leaves collected from almond trees closest to the poultry operation (AP 0 m) was more than 2-fold greater than from trees 120 m into the orchard or in the control orchards (Fig. 1). The 0-m row of each of the three orchards bordered dirt access roads for the orchard and surrounding growing environments (Fig. 5). Although not always significant, greater levels of dry solids were collected from AP at 0 m than from any other rows from AP or the control orchards. While an imperfect control, the data suggest that the higher dry solid levels in AP 0 m were not due just to proximity to the road.

More convincing evidence of movement of microorganisms from the poultry operation into AP came from examination of the relative abundance of the bacterial community members at the phylum and family levels. In AP at 120 m and all rows from AC1 and AC2, the phylum levels were remarkably similar, suggesting a baseline community. The four most dominant phyla accounted for over 95% of the community on average, and for these rows each of these phyla grouped statistically the same. However, with a single exception (*Bacteroidetes* for AC2 at 60 m) the community in AP at 0 m had a significantly different abundance of the dominant phyla. The abundance of the four dominant phyla in AP at 60 m was midway between AP at 0 m and the baseline from all other rows in AP and control orchards. The same phenomenon is evident at the family level with the top three bacterial families, which accounted for 42% of the relative abundance of the community on average; AP at 120 m and the control orchard rows grouped together to create a baseline abundance that AP at 0 m was significantly distinct from, with AP at 60 m in between (Fig. 3). These results suggest that as air moves from the outer to the inner rows of the orchard, dust particles are trapped by the orchard trees.

The family *Staphylococcaceae* was rarely identified in the control orchards but was the second most abundant bacterial family in the AP 0-m phyllosphere. As with the dominant phyla and families, AP 0-m communities were significantly distinct, AP 120-m communities were more similar to those in the control orchard rows, and AP 60-m communities were in between (Fig. 3 and 4). Members of *Staphylococcaceae* are common constituents of the chicken microbiome (39–42). It is unknown why the family *Staphylococcaceae* was the only member of the chicken microbiota that influenced AP at 0 m to a measurable degree. *Staphylococcaceae* have been identified in a variety of phyllospheres (43–45), and it may be that *Staphylococcaceae* survive better in the dry almond orchard environment. The presence of *Staphylococcaceae* in orchard air samples suggests air as a possible means of ingress into the orchard. The abundance of *Staphylococcaceae* in air samples from AP at 0 m in July 2013 was three times higher than any other time, which corresponded to the highest abundance of *Staphylococcaceae* in the phyllosphere sampled at the same time.

Required setback distances from the edge of concentrated animal feeding operations (e.g., >1,000 cattle or >82,000 poultry layers) to leafy green plantings were increased in 2018 from 120 m (400 ft) to 370 m (1,200 ft) for California and Arizona leafy greens growers (25, 26). A multitude of potentially influential risk factors were noted, including temperature, humidity, precipitation, and wind. The results of the current study provide evidence that airborne material and microorganisms could move a short distance, approximately 60 m (200 ft), into a downwind almond orchard that was 35 m (110 ft) from a commercial poultry operation with an estimated 60,000 poultry layers. Current almond harvest practices may lead to further distribution of these organisms. No evaluation of orchards that were upwind or to the sides of the poultry operation was made. The microbiome of the orchard phyllosphere was significantly altered, with the greatest impact occurring in the rows of the orchard closest to the poultry operation and the effects greatly reduced at distances 60 to 120 m into the orchard, possibly a result of the closest rows of trees providing a natural windbreak.

## MATERIALS AND METHODS

### Orchards and sampling.

Three commercial, conventional almond orchards located in the northern part of the San Joaquin Valley, California, were sampled over a 2-year period that coincided with a lengthy statewide drought that ran from 2011 to 2019. In the second year of the study, California received the least precipitation on record (46). “Almond Poultry” (AP) was a 23-year-old orchard of Delhi loamy sand soil adjacent to (first row of trees 35 m away from the poultry houses) and directly east (downwind) of a commercial egg-laying operation consisting of three houses of approximately 3,000 m^2^ each. Two orchards not near (>500 m from) animal agriculture were used as controls: “Almond Control 1” (AC1; 23 years old, 3.3 km from AP, Tinnin course sand soil) and “Almond Control 2” (AC2; 16 years old, 0.5 km from AP, Honcut sandy loam soil) (47). All three orchards were planted in a hedgerow formation, where the space between trees within rows is less than the space between rows and the between-row spaces are wide enough to allow access for equipment such as the mechanical shakers and sweepers used during harvest. The rows within each orchard were approximately 6.5 m apart, alternating between one of two or three almond varieties; no cover crops were used within the rows. AP was more densely planted; trees within rows were spaced approximately 4.6, 5.8, and 5.8 m apart for AP, AC1, and AC2, respectively. Air, tree (phyllosphere), drag swab, and soil samples were collected in each orchard; sampling within the poultry operation was not permitted. AP was sampled seven times in 2013, with six monthly samplings between March (when the trees bloomed and buds were pollinated) and August (when the fruit matured and hulls split); a single final sampling was conducted in mid-harvest. Mid-harvest samples were obtained after the first variety within the orchard had been shaken and those almonds had been windrowed, dried, and picked up. Samples were collected from AC1 on five occasions in 2013: the first four samplings occurred monthly between May and August, within 24 h of the corresponding AP samplings, and the fifth sampling occurred during mid-harvest. AP, AC1, and AC2 were sampled twice in 2014, with both samplings occurring on the same day in the three orchards in June and July prior to harvest.

### Sampling locations.

Grid layouts for the orchards were used to determine locations for sampling. In AP and AC1 the rows were planted in a north-south orientation; in AC2 the rows were planted in an east-west orientation. The predominant wind direction was from the west-northwest. Wind data from the closest California Irrigation Management Information System (CIMIS) weather station (Station 70, <15 km from all orchards) during the months sampled showed the median heading as 302°, with the first and third quartiles at 275° and 321°, respectively. The median of the average hourly wind speed was 2.2 m/s, with the first and third quartiles at 2.9 and 1.5 m/s, respectively. The maximum average hourly wind speed recorded during this period was 7.8 m/s. The first row of trees (closest to the poultry operation) was designated 0 m, and rows at 60 and 120 m into the orchard were identified. Three evenly separated points (A, B, and C) along each row were selected, for a total of nine sampling locations in each orchard (Fig. 5). Air, tree, and soil samples were taken at all nine sample locations, except in 2014, when air samples were collected at only the six locations from the outermost columns (i.e., A and C). Before each sampling, atmospheric data, including wind speed, temperature, and relative humidity, were recorded at each location within the orchards by using a Kestrel 4500 pocket weather tracker (Nielsen-Kellerman, Boothwyn, PA).

### Leaf, fruit, and twig sample collection.

Samples, collected randomly from multiple trees at each location, consisted of twigs that typically had leaves along with developing fruit. Samples were collected by breaking twigs off the tree branches by hand into freshly opened zip bags (41 by 41 cm) and using fresh nitrile gloves at each of the nine sampling locations. Samples were transported to the laboratory on ice and processed within 24 h of collection. In the laboratory, samples were rinsed using two different methods. To extract DNA for sequencing to determine the phyllosphere composition, 100 g was weighed into a 1,600-ml Whirl-Pak filter bag (Nasco, Modesto, CA), and 100 ml of 0.01 M K_2_PO_4_ buffer supplemented with 0.5 ml/liter of Tween 20 (Sigma-Aldrich, St. Louis, MO) was added. Each bag was vigorously shaken for 30 s and then sonicated for 15 min in a Branson 8510 ultrasonic cleaner water bath (Branson Ultrasonics Corp., Danbury, CT). The resulting mixture of buffer and dust was evenly pipetted into two 50-ml tubes and centrifuged at 8,228 × *g* for 10 min. The supernatant was poured off and the resulting pellet was stored at −20°C until DNA was extracted. Unless otherwise indicated, all media were obtained from BD, Franklin Lakes, NJ.

To obtain the dust for dry solid measurement, petioles (leaf stems) were first removed and the leaves (25.0 ± 0.2 g) alone were shaken with 75.0 ± 0.1 ml of ultrapure water (Milli-Q Advantage A10; MilliporeSigma, Burlington, MA) in 1,600-ml Whirl-Pak bags. The leaf rinsate (10.0 ± 0.1 ml) was pipetted into a preweighed aluminum dish, and samples were heated in a 100°C oven for approximately 3 h, until all moisture was evaporated, and then cooled to ambient temperature and weighed.

### Drag swabs.

Drag swabs were collected along the orchard rows within the orchards in all years. Drag swabs were prepared as described previously (35). Briefly, the drag swabs, which resemble a bowtie, were made by tightly tying the end of a 1-m piece of butcher’s string around the center of a 12-ply sterile gauze pad (10 by 10 cm; CVS, Woonsocket, RI). Prepared swabs were autoclaved and then transferred aseptically to individual 1,600-ml Whirl-Pak bags, leaving a 10-cm length of string outside the bag (to aid in aseptic removal of the swab in the field). Sterile, nonfat evaporated milk (12 ml; Nestle, Solon, OH) was added aseptically to each bag and allowed to soak into the swab before the swabs were frozen and stored at −20°C. Swabs were thawed and then refrigerated (4 ± 2°C) for up to 1 day prior to use. In the orchard, the swabs were dragged along each of the designated sampling rows before any other orchard sampling took place. Drag speed was based on typical adult walking speed. Four drag swabs were pooled into a single sample for each of the three designated rows in an orchard. Pooled samples were transported on ice, refrigerated, and, within 24 h, submitted to the California Animal Health and Food Safety Laboratory System (Davis, CA) for *Salmonella* multistep enrichment, as described by Uesugi et al. (35), with the addition of PCR screening to identify presumptive positives.

### Soil samples.

Four scoops of topsoil (∼3 cm deep) were removed from each of the nine locations within each orchard. In 2013, two additional soil samples were collected near drip line outlets. Before any additional treatment, a portion of soil taken from each location was reserved for DNA extraction. From the remaining sample, 100 g of pooled soil was combined in a 530-ml Whirl-Pak filter bag with 200 ml of 0.02 M Na_2_PO_4_ buffer supplemented with Tween 20 (0.5 ml/liter), and the bag was shaken vigorously for 30 s. About 100 ml of filtrate was then added to 100 ml of universal preenrichment broth (2× UPB) in a 530-ml Whirl-Pak filter bag and incubated for 18 ± 2 h at 37 ± 2°C. Recovery of E. coli, other coliforms, or *Salmonella* from these enrichments is described below.

### Air samples.

Air samples were collected with MAS-100 Eco samplers (EMD Millipore, Billerica, MA). With the sampler, air is pulled through the unit, resulting in the impaction of airborne microorganisms onto an agar plate. Samplers were run at their maximum setting, 100 liters/min, to collect the greatest number of airborne bacteria for selective sample enrichment. In 2013 (at nine orchard locations) and 2014 (at six orchard locations), air samples were collected in triplicate using MAS-100 Eco samplers. Samplers were run for 10 min with plates of 20% Reasoner’s 2A agar (R2A; Oxoid, Basingstoke, UK) supplemented with pyruvic acid at 1 g/liter to assist in cell repair of injured or desiccated bacterial cells. In the 2014 sampling, an additional 1.5 g/liter of agar was included (total, 2.5 g/liter) to increase the robustness of the plates for transportation to the laboratory. Samples were incubated at 25 to 35°C during transport to allow cell repair and then held at 35°C for 18 ± 3 h to complete the incubation. Colonies were enumerated according to the sampler instructions, and then the agar in each petri dish was dissolved in 50 ml of buffered peptone water (BPW; Oxoid) and incubated for 6 ± 1 h at 35 ± 2°C. The BPW enrichment (10 ml) was transferred to a tube containing 90 ml of double-strength UPB and incubated for 15 ± 2 h at 37 ± 2°C. This process was developed to allow further bacterial cell repair of injured or desiccated microorganisms. In 2013, a portion of the UPB enrichment was reserved for DNA extraction and subsequent culture-independent analysis. The presence or absence of E. coli, other coliforms, or *Salmonella* was determined as described below.

### Recovery of E. coli, other coliforms, or *Salmonella* from air and soil enrichments.

The UPB enrichments were streaked onto ChromECC (CHROMagar, Paris, France) for detection of E. coli (blue colonies) and other coliforms (mauve colonies). In 2013 only, for recovery of *Salmonella*, a portion of the UPB enrichment (10 ml) was also transferred to 90 ml of tetrathionate broth base supplemented with iodine at 20 ml/liter and then incubated for 6 ± 1 h at 42 ± 2°C. The tetrathionate broth base enrichment (10 ml) was transferred to 90 ml of mBroth (Biocontrol Systems, Inc., Bellevue, WA), incubated for 12 ± 1 h at 37 ± 2°C, streaked onto XLT4 agar, and then incubated at 37 ± 2°C for 24 ± 2 h for detection of *Salmonella*.

### DNA extraction, 16S rRNA gene amplification, and sequencing library construction.

The phyllosphere, enriched air, and soil microbiota collected from AP and AC1 in July, August, and mid-harvest in 2013 and the phyllosphere and soil microbiota collected from AP, AC1, and AC2 in 2014 were identified and analyzed by culture-independent second-generation sequencing. Briefly, genomic DNA (gDNA) from the air samples was extracted and purified from the air-UPB enrichments using the PowerFood kit from Mo Bio (Carlsbad, CA) according to the manufacturer’s recommendations. For the phyllosphere and soil samples, gDNA was extracted and purified using the Mo Bio PowerSoil kit according to the manufacturer’s recommendations. Both kits include a bead-beating step that occurred on a Vortex Genie 2 (Scientific Industries, Inc., Bohemia, NY) set to maximum rotation (3,200 rpm) in a Mo Bio vertical adapter. The V4 domain of bacterial 16S rRNA gene was amplified from the purified gDNA, as described previously (48). Briefly, PCR was performed with forward primer F515 (5′-*NNNNNNNN*GTGTGCCAGCMGCCGCGGTAA-3′) with an 8-bp barcode sequence (italicized) and 2-bp linker sequence (underlined) on the 5′ end and reverse primer R806 (5′-GGACTACHVGGGTWTCTAAT-3′) (49) and the following PCR conditions: 95°C for 2 min and then 30 to 35 cycles of 95°C for 30 s, 55°C for 30 s, and 72°C for 30 s, with a final step at 72°C for 5 min. The barcoded 16S rRNA gene amplicons were pooled into an equimolar single sample and submitted for library construction and paired-end DNA sequencing using the Illumina MiSeq platform at the UC Davis Genome Center (Davis, CA).

### Data processing and analysis.

The 16S rRNA gene sequences were quality filtered and processed with the Quantitative Insights into Microbial Ecology (QIIME) software package (50). The sequences were demultiplexed according to the unique 8-bp barcode sequence associated with each sample and were filtered to remove low-quality reads; those sequences containing windows of 50 bp with an average quality score less than 30 were truncated at the beginning of the low-quality section. If either a truncated or nontruncated sequence was less than 200 bp in length after barcode and primer trimming, that sequence was removed from the data set. The 16S rRNA gene sequences were assigned to OTU using UCLUST (51) at the 97% similarity level, and then a representative sequence from each OTU was assigned a taxonomic identity with the GreenGenes database version 13_5 (52). Representative sequences for each OTU were aligned in QIIME using the PyNast algorithm (53), and phylogenetic trees of the assigned OTU were created using FastTree (54). The alpha-diversity metrics Chao1 and Shannon index were used to compare the diversity of bacteria in the microbiome. For beta-diversity comparisons, analysis of similarity (ANOSIM) was calculated with 999 permutations using both the unweighted and weighted UniFrac metric (55). Principal-coordinate analysis was used to compare the total diversity differences among samples, with the jackknifing parameter enabled for 10 iterations. Alpha-diversity and beta-diversity comparisons were performed in QIIME with a depth of 1,500 sequences and 10 iterations; rarefaction curves can be found in Fig. S6. Differences among taxonomic abundances were determined using analysis of variance (ANOVA), and means were compared using Tukey’s honestly significant difference (HSD) test. Differences in proportions of samples positive for E. coli and other coliforms were determined using the chi-square test. These statistics were performed with JMP Pro 14 software (SAS Institute, Cary, NC), and differences were considered significant at a *P* value of <0.05.

### Data availability.

Sequencing data are available on Qiita, study identifier 13151 (https://qiita.ucsd.edu/study/description/13151).

## Supplementary Material

Supplemental file 1
