# Supplementary material for: Microorganisms Move a Short Distance into an Almond Orchard from an Adjacent Upwind Poultry Operation
Source: Appl Environ Microbiol. 2020 Jul 20;86(15):e00573-20. doi: 10.1128/AEM.00573-20 (PMC7376559; doi:10.1128/AEM.00573-20)
Supplement: Supplemental file 1 [file AEM.00573-20-s0001.pdf]

Table S1. Wind speed (m/s), temperature (°C), and relative humidity (%) averaged across sampling locations in almond orchards[a] in 2013 and 2014.

Orchard sampling period	Wind Speed (m/s)			Temperature (°C)			Relative Humidity (%)		
	AP	AC1	AC2	AP	AC1	AC2	AP	AC1	AC2
**2013**									
March	0.0 ± 0.0	ND[b]	ND	15.3 ± 0.3	ND	ND	0.63 ± 0.03	ND	ND
April	0.5 ± 0.4	ND	ND	16.1 ± 0.3	ND	ND	0.73 ± 0.00	ND	ND
May	0.3 ± 0.2	0.2 ± 0.2	ND	25.8 ± 1.3	20.8 ± 0.5	ND	0.45 ± 0.03	0.54 ± 0.03	ND
June	1.2 ± 0.8	1.7 ± 1.7	ND	22.0 ± 1.5	ND	ND	0.50 ± 0.02	0.43 ± 0.01	ND
July	1.0 ± 0.8	0.6 ± 0.7	ND	21.1 ± 1.0	28.9 ± 1.3	ND	0.62 ± 0.02	0.41 ± 0.02	ND
August	0.6 ± 0.3	0.8 ± 0.4	ND	16.5 ± .21	31.3 ± 0.4	ND	0.76 ± 0.01	0.41 ± 0.02	ND
Mid-harvest	0.7 ± 0.7	0.8 ± 0.5	ND	23.9 ± 1.4	29.5 ± 2.0	ND	0.31 ± 0.02	0.34 ± 0.04	ND
**2014**									
June	0.5 ± 0.3	0.9 ± 0.5	0.6 ± 0.3	29.1 ± 1.4	30.9 ± 0.5	26.8 ± 2.5	0.38 ± 0.01	0.34 ± 0.03	0.41 ± 0.05
July	1.3 ± 0.8	0.7 ± 0.3	0.7 ± 0.2	27.0 ± 2.1	32.6 ± 1.7	27.1 ± 2.2	0.53 ± 0.05	0.44 ± 0.03	0.55 ± 0.04

[a] Orchard designations: AP, Almond Poultry; AC1, Almond Control 1; AC2, Almond Control 2.

[b] ND, not determined; AC1 not sampled until May 2013, and AC2 not sampled until 2014.

Table S2. Distribution of microbial populations in air by aerobic plate count (log CFU/m^3 air) and by enrichment and isolation results (*E. coli*, other coliforms) for samples from almond orchards by row (0 m, 60 m, 120 m) and column (A, B, C) in 2013, *n* = 3.

		Air sample aerobic plate count (log CFU/m^3 air) and enrichment results (*E*, *C*)[a]					
		Orchard AP			Orchard AC1		
		Column			Column		
Month	Row	A	B	C	A	B	C
	0 m	2.3 *C*	2.4 *C*	2.1 *C*	ND[b]	ND	ND
March	60 m	2.3 *C*	2.7 n	2.0 *C*	ND	ND	ND
	120 m	2.3 n	2.3 n	2.0 n	ND	ND	ND
	0 m	1.3 *E,C*	1.2 *E,C*	1.5 *E,C*	ND	ND	ND
April	60 m	1.1 *E,C*	1.2 *E,C*	1.5 *C*	ND	ND	ND
	120 m	1.2 *E*	1.0 *E*	1.6 *E,C*	ND	ND	ND
	0 m	2.4 *C*	2.4 *C*	2.3 *C*	2.1 *C*	2.2 *C*	2.3 *C*
May	60 m	2.2 *C*	2.3 *C*	2.4 *C*	2.2 *C*	2.2 *C*	2.4 *C*
	120 m	2.2 *C*	2.2 *C*	2.2 *C*	2.1 *C*	2.2 *C*	2.1 *C*
	0 m	2.7 *E,C*	2.4 *E,C*	2.4 *E,C*	>3.4[c] *C*	>3.4 *C*	>3.4 *C*
June	60 m	2.5 *E,C*	2.3 *C*	2.4 *C*	>3.4 *C*	>3.4 *C*	>3.4 *C*
	120 m	2.9 *C*	2.3 *E,C*	2.4 *E,C*	>3.4 *C*	>3.4 *C*	>3.4 *C*
	0 m	3.0 *E*	>3.4 *C*	3.3 n	2.5 *C*	2.5 *C*	2.5 *C*
July	60 m	>3.4 *C*	3.1 n	3.0 *C*	2.6 *C*	2.5 *C*	2.6 *C*
	120 m	2.7 *C*	2.5 *C*	2.4 *C*	2.4 *C*	2.6 *C*	2.6 *C*
	0 m	3.0 *C*	2.6 *C*	NC[d]	2.3 n	2.5 *C*	2.3 *C*
August	60 m	2.9 *C*	2.9 *C*	NC	2.2 *C*	2.2 *C*	2.2 *C*
	120 m	2.7 *C*	2.4 *C*	NC	2.4 *C*	2.3 *C*	2.4 *C*
	0 m	>3.4 *E,C*	>3.4 *E,C*	>3.4 *C*	>3.4 *C*	>3.4 *C*	>3.4 *C*
Mid-harvest	60 m	>3.4 *C*	>3.4 *C*	>3.4 *C*	>3.4 *C*	>3.4 *C*	>3.4 *C*
	120 m	>3.4 *C*	>3.4 *C*	>3.4 *C*	>3.4 *C*	>3.4 *C*	3.2 *C*

[a] Within cells, letters after aerobic plate count indicate presence of *E. coli* (*E*), other coliforms (*C*), or negative (n) results after enrichment and isolation on ChromECC.

[b] ND, not determined; AC1 not sampled until May 2013, and AC2 not sampled until 2014.

[c] Upper limit of detection (ULOD) was 3.4 log CFU/m^3 air.

[d] NC, sample not collected.

Table S3. Distribution of microbial populations in air by aerobic plate count (log CFU/m^3 air) and by enrichment and isolation results (*E. coli*, other coliforms) for samples from almond orchards by row (0 m, 60 m, 120 m) and column (A, C) in 2014, n = 3

		Microbial population in air (log CFU/m^3)					
		Orchard AP		Orchard AC1		Orchard AC2	
		Column		Column		Column	
Month	Row	A	C	A	C	A	C
June	0 m	>3.4 *E*,*C* [a,b]	>3.4 *E*,*C*	>3.4 *C*	>3.4 *C*	3.3 *C*	3.2 *C*
	60 m	>3.4 *C*	>3.4 *C*	>3.4 *C*	>3.4 *C*	3.2 *C*	3.3 *C*
	120 m	>3.4 *C*	>3.4 *C*	>3.4 *C*	>3.4 n	2.6 *C*	3.0 *E*
July	0 m	>3.4 *E*,*C*	>3.4 *C*	3.2 *C*	>3.4 *C*	>3.4 *C*	3.2 *C*
	60 m	>3.4 *E*,*C*	>3.4 *C*	>3.4 *C*	3.0 *C*	>3.4 n	>3.4 *C*
	120 m	>3.4 *C*	>3.4 *C*	>3.4 *C*	2.8 *C*	>3.4 *C*	>3.4 *C*

[a] Within cells, letters after aerobic plate count indicate presence of *E. coli* (*E*), other coliforms (*C*), or negative (n) results after enrichment and isolation on ChromECC.
[b] ULOD was 3.4 log CFU/m^3.

Table S4. Dominant bacterial phyla in the orchard microbial community of three almond orchards (AP, AC1, and AC2) as determined by second-generation sequencing

		Relative abundance (%) of dominant bacterial phyla in orchard samples at sampling times														
		AP						AC1						AC2		
Orchard sample	Bacterial phylum	July 2013	Aug 2013	Sept 2013	June 2014	July 2014	Avg	July 2013	Aug 2013	Sept 2013	June 2014	July 2014	Avg	June 2014	July 2014	Avg
Soil	*Actinobacteria*	31.84	22.77	27.63	23.89	24.60	26.15[A][a]	29.99	21.62	23.88	19.89	16.92	22.46[B]	21.42	26.56	23.99[AB]
	Bacteroidetes	7.33	5.13	7.22	9.29	9.01	7.60	5.74	4.07	5.45	9.28	12.04	7.316	6.65	5.96	6.31
	Chloroflexi	6.68	14.39	5.92	5.95	6.16	7.82[B]	9.19	15.36	15.04	8.18	7.50	11.05[A]	12.37	7.62	10.00[AB]
	Firmicutes	7.42	5.97	13.50	8.26	11.30	9.29[A]	4.31	7.21	4.22	5.15	4.80	5.138[B]	7.99	9.41	8.70[A]
	Proteobacteria	29.91	21.49	32.66	32.01	30.33	29.28	31.68	21.31	29.16	32.57	34.49	29.84	26.94	34.33	30.64
Phyllo-sphere	*Actinobacteria*	21.06	29.39	25.6	22.21	15.34	22.72[A]	10.69	13.40	20.3	11.95	8.92	13.05[B]	15.80	9.98	12.89
	Bacteroidetes	15.90	5.48	15.3	11.88	17.68	13.25[B]	21.33	13.23	11.7	22.69	23.51	18.49[B]	15.96	23.11	19.54
	Chloroflexi	0.40	0.90	0.7	0.79	0.76	0.71[B]	0.27	1.14	2.60	1.42	0.73	1.232	0.82	0.74	0.78
	Firmicutes	23.33	41.54	8.10	27.36	19.99	24.06[A]	6.58	23.11	8.10	10.43	7.38	11.12	5.31	7.60	6.46
	Proteobacteria	36.04	20.83	45.6	34.94	42.83	36.05[B]	55.91	46.48	52.4	50.70	56.66	52.43	58.46	55.36	56.91
Air	*Actinobacteria*	0.48	1.06	0.90	ND[b]	ND	0.81[A]	0.46	0.48	0.70	ND	ND	0.54	ND	ND	
	Bacteroidetes	0.07	0.07	0.20	ND	ND	0.11	0.09	0.08	0.20	ND	ND	0.12	ND	ND	
	Chloroflexi	0.06	0.06	0.10	ND	ND	0.07	0.07	0.07	0.10	ND	ND	0.08	ND	ND	
	Firmicutes	61.03	65.67	25.90	ND	ND	50.87	49.20	65.37	21.20	ND	ND	45.25	ND	ND	
	Proteobacteria	38.27	33.06	72.60	ND	ND	47.98	50.06	33.92	77.60	ND	ND	53.86	ND	ND	

[a] Within rows for orchard sample type, phylum prevalence averages with different uppercase letters are significantly different ($P < 0.05$).

[b] ND, not determined.

Table S5. Dominant bacterial families in the orchard microbial community of three almond orchards (AP, AC1, and AC2) as determined by second-generation sequencing

Orchard sample	Bacterial family	Relative abundance (%) of dominant bacterial families in orchard samples at sampling times														
		AP						AC1						AC2		
		July 2013	Aug 2013	Sept 2013	June 2014	July 2014	Avg	July 2013	Aug 2013	Sept 2013	June 2014	July 2014	Avg	June 2014	July 2014	Avg
Soil	*Bacillaceae*	1.34	1.04	6.48	5.34	7.45	4.33^{Aa}	1.68	3.19	2.80	3.35	3.01	2.81^{B}	4.80	5.33	5.07^{A}
	Cytophagaceae	1.94	1.94	2.61	2.41	1.98	2.18^{B}	2.27	1.43	2.42	3.18	4.31	2.72^{A}	2.74	2.43	2.59^{AB}
	Entero-bacteriaceae	0.72	0.69	2.14	0.18	0.19	0.78^{A}	1.04	0.67	1.47	0.19	0.18	0.71^{AB}	0.22	0.34	0.28^{B}
	Methylo-bacteriaceae	0.32	0.20	0.59	0.95	0.94	0.60^{A}	0.18	0.15	0.35	0.75	0.70	0.43^{B}	0.49	0.62	0.56^{AB}
	Micro-bacteriaceae	1.33	0.87	1.73	0.50	0.76	1.04^{A}	1.00	0.60	0.97	0.74	0.58	0.78^{B}	1.18	1.15	1.17^{B}
	Micrococcaceae	9.98	4.49	7.64	6.36	7.07	7.11^{A}	4.03	4.09	3.45	4.51	4.16	4.05^{B}	6.14	7.45	6.80^{A}
	Planococcaceae	3.32	2.20	2.05	0.06	0.07	1.54^{A}	1.34	2.07	0.78	0.42	0.40	1.00^{B}	1.00	0.99	1.00^{B}
	Pseudo-monadaceae	0.84	0.39	1.24	0.41	0.28	0.63^{A}	0.40	0.31	0.71	0.33	0.41	0.43^{B}	0.43	0.50	0.47^{AB}
	Sphingo-monadaceae	3.99	2.19	3.65	8.85	10.29	5.79^{AB}	3.34	2.00	3.12	8.08	7.13	4.73^{B}	6.38	8.39	7.39^{A}
	Staphylo-coccaceae	0.23	0.07	.0023	0.30	0.36	0.19^{A}	0.18	0.06	.0005	0.21	0.20	0.13^{B}	0.24	0.33	0.29^{A}
Phyllo-sphere	*Bacillaceae*	6.12	13.67	3.07	11.57	8.61	8.61	1.60	6.85	4.73	4.76	3.63	4.31	2.66	3.91	3.29
	Cytophagaceae	12.84	4.70	13.07	9.04	13.69	10.67	17.18	11.47	9.60	18.82	19.98	15.41	13.03	18.46	15.75
	Entero-bacteriaceae	1.17	2.72	3.12	2.62	0.76	2.08	1.64	6.70	4.85	2.40	4.83	4.08	2.20	2.40	2.30
	Methylo-bacteriaceae	7.02	3.77	10.49	4.93	6.73	6.59	13.23	9.37	11.19	6.97	10.66	10.28	11.68	11.27	11.48
	Micro-bacteriaceae	3.38	2.53	7.29	2.82	2.87	3.78	3.61	3.10	3.49	3.64	2.58	3.28	4.35	2.96	3.66
	Micrococcaceae	0.78	1.41	3.07	1.12	1.06	1.49	0.48	1.27	3.00	1.20	0.77	1.34	1.04	0.88	0.96
	Planococcaceae	1.31	2.95	0.71	0.68	0.83	1.30	1.41	4.83	0.97	0.81	0.73	1.75	0.48	0.70	0.59
	Pseudo-monadaceae	1.89	1.30	1.61	2.90	1.39	1.82	1.44	2.85	1.33	2.76	1.50	1.98	3.48	0.82	2.15

	Sphingo-monadaceae	14.49	6.83	18.39	15.92	22.37	15.60	22.57	15.56	16.58	23.35	25.88	20.79	27.41	25.16	26.29
	Staphylo-coccaceae	7.46	8.81	2.70	7.25	4.00	6.04	0.21	0.79	.0013	0.49	0.48	**0.39**	0.35	0.41	0.38
Air	*Bacillaceae*	15.17	8.86	8.42	ND[b]	ND	10.82	3.40	3.85	8.13	ND	ND	5.13	ND	ND	
	Cytophagaceae	0.05	0.06	0.12	ND	ND	0.08	0.07	0.06	0.14	ND	ND	0.09	ND	ND	
	Entero-bacteriaceae	31.30	21.13	57.64	ND	ND	36.69	40.49	26.60	52.82	ND	ND	39.97	ND	ND	
	Methylo-bacteriaceae	0.06	0.05	0.12	ND	ND	0.08	0.05	0.05	0.13	ND	ND	0.08	ND	ND	
	Micro-bacteriaceae	0.03	0.03	0.09	ND	ND	0.05	0.03	0.03	0.09	ND	ND	0.05	ND	ND	
	Micrococcaceae	0.15	0.78	0.21	ND	ND	0.38	0.11	0.17	0.22	ND	ND	0.17	ND	ND	
	Planococcaceae	9.22	7.92	11.18	ND	ND	9.44	8.17	6.01	13.50	ND	ND	9.23	ND	ND	
	Pseudo-monadaceae	2.72	4.38	15.26	ND	ND	7.45	8.27	3.45	15.10	ND	ND	8.94	ND	ND	
	Sphingo-monadaceae	0.12	0.10	0.34	ND	ND	0.19	0.12	0.10	0.36	ND	ND	0.19	ND	ND	
	Staphylo-coccaceae	2.65	0.95	.001	ND	ND	1.20	0.16	0.68	.01	ND	ND	0.28	ND	ND	

[a] Within rows for orchard sample type, family prevalence averages with different uppercase letters are significantly different ($P < 0.05$).

[b] ND, not determined.

Table S6. Dominant bacterial genera in the orchard microbial community of three almond orchards (AP, AC1, and AC2) as determined by next-generation sequencing

		Relative abundance (%) of dominant bacterial genera in orchard samples at sampling times														
		AP						AC1						AC2		
Orchard sample	Bacterial genus	July 2013	Aug 2013	Sept 2013	June 2014	July 2014	Avg	July 2013	Aug 2013	Sept 2013	June 2014	July 2014	Avg	June 2014	July 2014	Avg
Soil	*Arthrobacter*	6.10	2.64	2.44	1.37	1.44	2.80^{Ba}	1.57	1.48	0.62	1.27	1.18	1.22^{C}	4.17	5.15	4.66^{A}
	Bacillus	0.87	0.61	6.04	4.81	7.22	3.91^{A}	0.74	0.82	1.98	3.00	2.68	1.84^{B}	4.07	4.37	4.22^{A}
	Balneimonas	2.25	2.23	1.81	2.06	1.91	2.05^{B}	2.77	1.73	2.66	2.59	3.34	2.62^{A}	2.24	2.69	2.47^{AB}
	Brevibacterium	0.07	0.03	0.01	0.06	0.07	0.05^{A}	0.03	0.02	0.001	0.02	0.02	0.02^{B}	0.03	0.03	0.03^{B}
	Erwinia	0.12	0.09	0.71	0.07	0.08	0.21	0.10	0.11	0.16	0.08	0.07	0.10	0.12	0.10	0.11
	Hymenobacter	0.17	0.08	0.25	0.53	0.50	0.31	0.12	0.08	0.16	0.69	0.68	0.35	0.40	0.42	0.41
	Methylo-bacterium	0.21	0.12	0.66	0.85	0.86	0.54	0.14	0.11	0.30	0.68	0.63	0.37	0.46	0.58	0.52
	Skermanella	2.38	1.28	1.82	0.90	0.88	1.45^{B}	3.31	2.48	3.39	1.61	2.01	2.56^{A}	0.73	0.96	0.85^{B}
	Sphingomonas	1.26	0.72	0.57	1.58	1.58	1.14	1.15	0.68	0.51	1.42	1.27	1.01	1.09	1.37	1.23
	Staphylococcus	0.12	0.05	0.18	0.07	0.10	0.10^{A}	0.08	0.05	0.03	0.04	0.05	0.05^{B}	0.05	0.14	0.10^{AB}
Phyllo-sphere	*Arthrobacter*	0.29	0.42	0.35	0.39	0.34	0.36	0.14	0.35	0.73	0.49	0.29	0.40	0.33	0.26	0.30
	Bacillus	0.93	2.30	1.07	2.24	2.80	1.87^{B}	1.11	4.75	4.11	4.09	3.01	3.41^{A}	2.19	3.32	2.76^{AB}
	Balneimonas	0.12	0.22	0.54	0.29	0.30	0.29^{B}	0.14	0.42	1.56	0.49	0.40	0.60^{A}	0.50	0.39	0.45^{AB}
	Brevibacterium	3.24	5.43	1.31	2.34	0.95	2.65^{A}	0.04	0.07	0.001	0.06	0.03	0.04^{B}	0.04	0.03	0.04^{B}
	Erwinia	0.46	1.89	1.16	1.56	0.44	1.10	0.81	2.35	1.05	1.38	4.30	1.98	1.86	0.93	1.40
	Hymenobacter	10.98	3.85	11.12	7.63	12.08	9.13^{B}	14.69	9.98	7.91	17.08	18.23	13.58^{A}	10.38	16.61	13.50^{A}
	Methylo-bacterium	7.00	3.75	10.44	4.91	6.72	6.56^{B}	13.21	9.34	11.09	6.95	10.64	10.25^{A}	11.67	11.27	11.47^{A}
	Skermanella	0.06	0.05	0.19	0.07	0.08	0.09^{B}	0.07	0.09	0.63	0.16	0.12	0.21^{A}	0.09	0.11	0.10^{A}
	Sphingomonas	13.80	6.45	13.06	11.38	16.51	12.24^{B}	21.06	14.59	11.35	17.41	19.06	16.69^{A}	20.70	18.19	19.45^{A}
	Staphylococcus	4.48	4.75	2.07	3.26	1.65	3.24^{A}	0.10	0.29	0.07	0.17	0.14	0.15^{B}	0.08	0.08	0.08^{B}
Air	*Arthrobacter*	0.07	0.67	0.07	ND[b]	ND	0.27	0.05	0.11	0.05	ND	ND	0.07	ND	ND	

Bacillus	13.89	8.64	7.73	ND	ND	10.09[A]	3.23	3.67	8.36	ND	ND	5.09[B]	ND	ND
Balneimonas	0.01	0.01	0.04	ND	ND	0.02	0.01	0.01	0.03	ND	ND	0.02	ND	ND
Brevibacterium	0.02	0.01	0.001	ND	ND	0.01	0.02	0.02	0.001	ND	ND	0.01	ND	ND
Erwinia	4.75	3.94	7.31	ND	ND	5.33	4.27	9.68	2.41	ND	ND	5.45	ND	ND
Hymenobacter	0.04	0.05	0.09	ND	ND	0.06	0.05	0.05	0.08	ND	ND	0.06	ND	ND
Methylo-bacterium	0.06	0.05	0.13	ND	ND	0.08	0.05	0.05	0.12	ND	ND	0.07	ND	ND
Skermanella	0.02	0.01	0.03	ND	ND	0.02	0.02	0.02	0.03	ND	ND	0.02	ND	ND
Sphingomonas	0.11	0.09	0.23	ND	ND	0.14	0.11	0.09	0.22	ND	ND	0.14	ND	ND
Staphylococcus	2.43	0.55	0.71	ND	ND	1.23[A]	0.12	0.13	0.06	ND	ND	0.10[B]	ND	ND

[a] Within rows for orchard sample type, genus prevalence averages with different uppercase letters are significantly different ($P < 0.05$).

[b] ND, not determined.

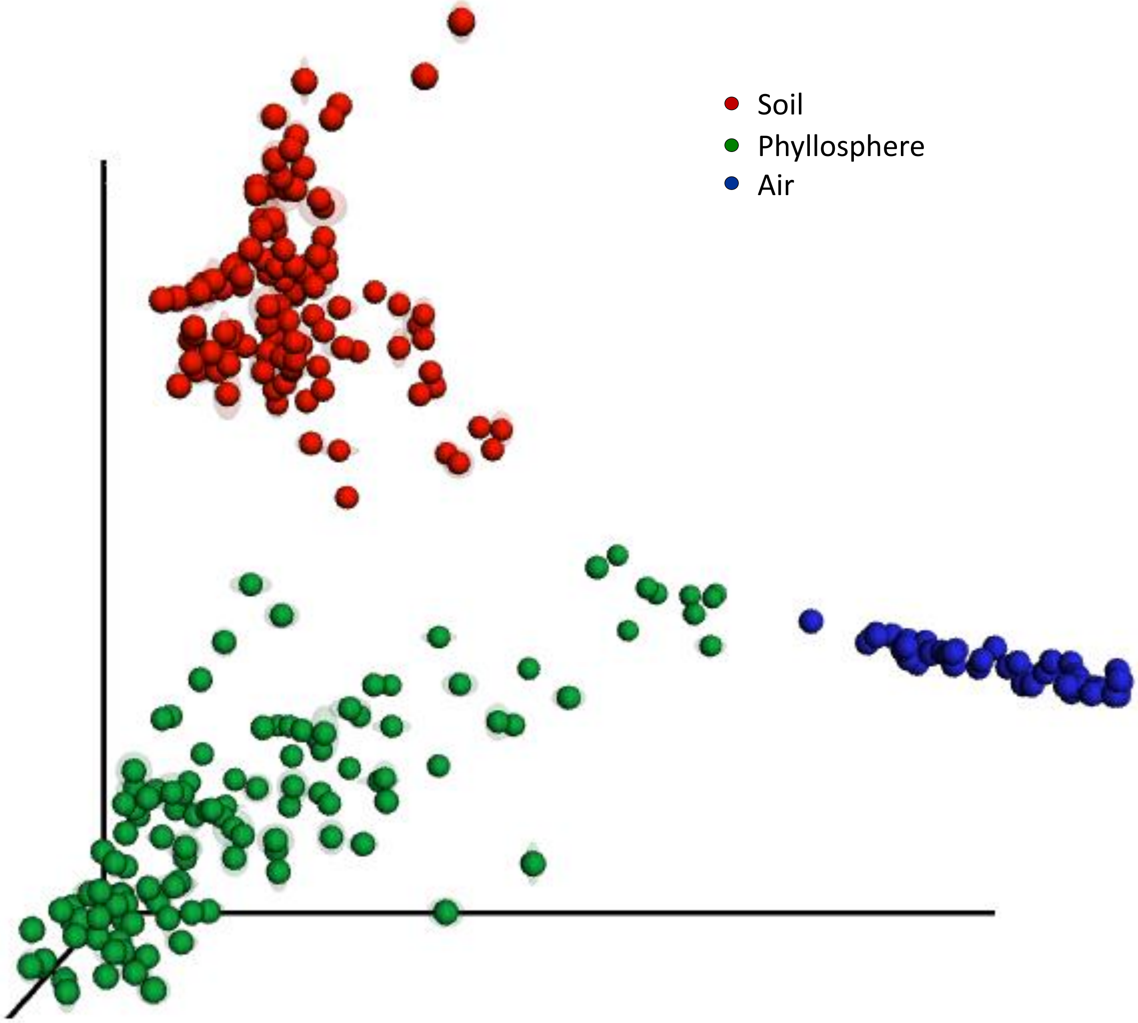

Figure S1. Principle coordinate analysis of almond orchard samples (soil, phyllosphere, air) using the weighted UniFrac community distance metric. Air, *n* = 50; Phyllosphere, *n* = 125; Soil, *n* = 113.

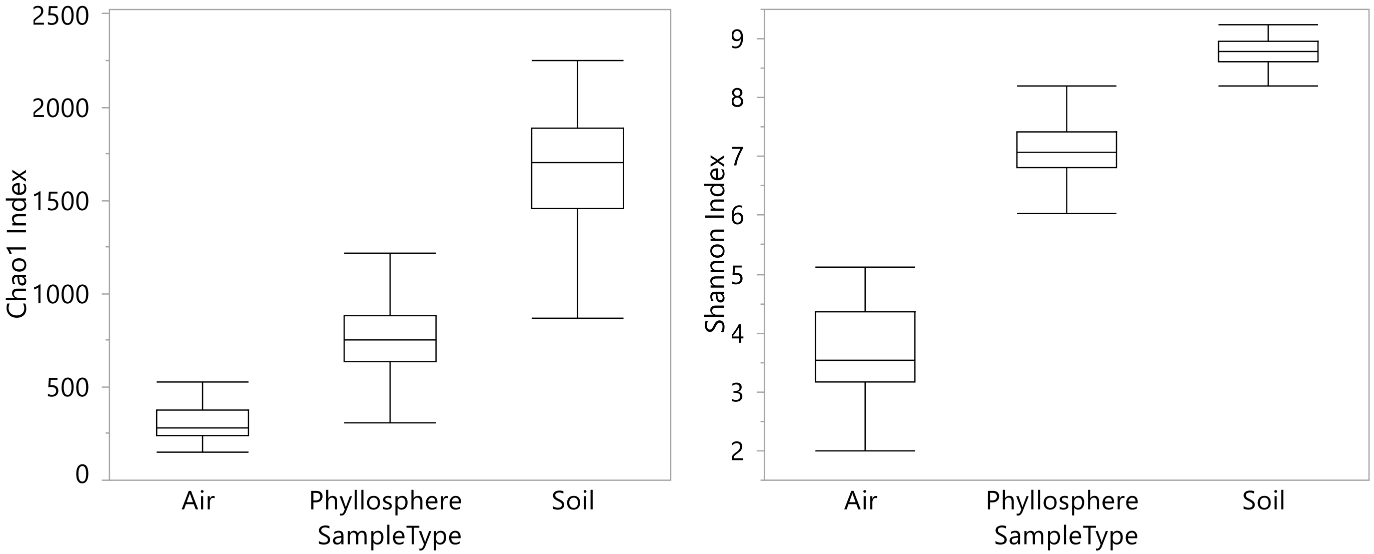

Figure S2. Box plots showing the alpha diversity of the microbial communities in air, phyllosphere, and soil samples as measured by Chao1 and Shannon index. Air: AP, *n* = 27; AC1, *n* = 23; AC2. Phyllosphere: AP, *n* = 63; AC1, *n* = 44; AC2, *n* = 18. Soil: AP, *n* = 50; AC1, *n* = 45; AC2, *n* = 18.

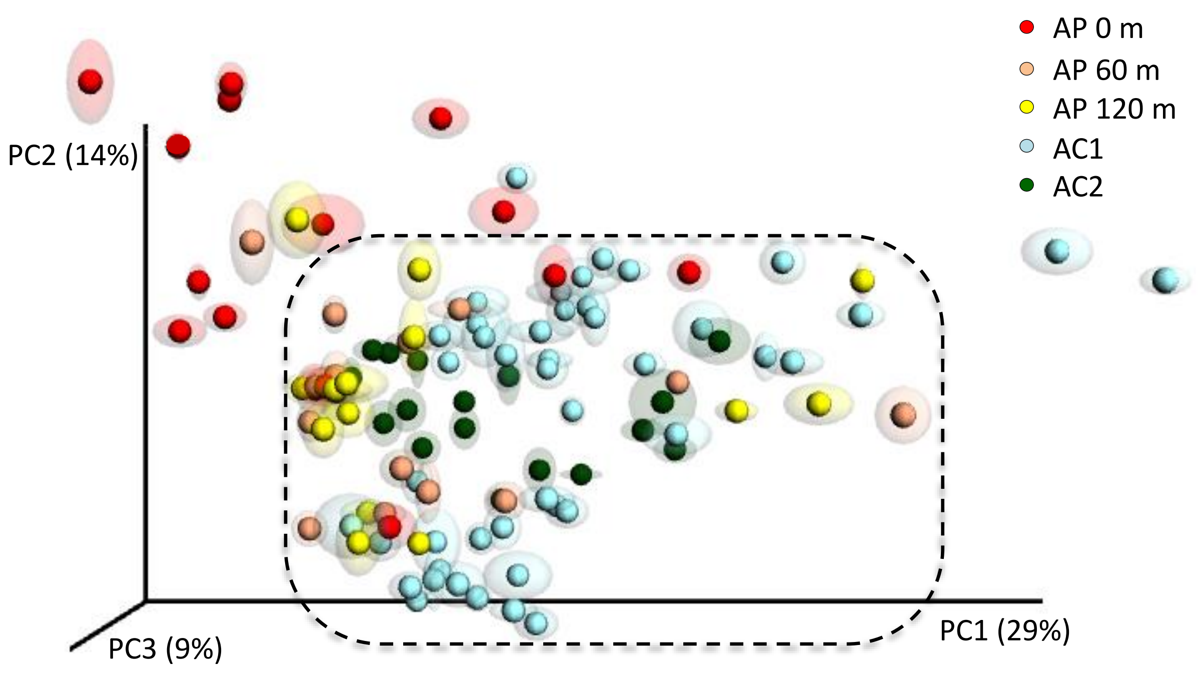

Figure S3. Principle coordinate analysis of almond orchard soil samples using the weighted UniFrac community distance metric. AP, *n* = 50; AC1, *n* = 45; AC2, *n* = 18.

A. Phyla

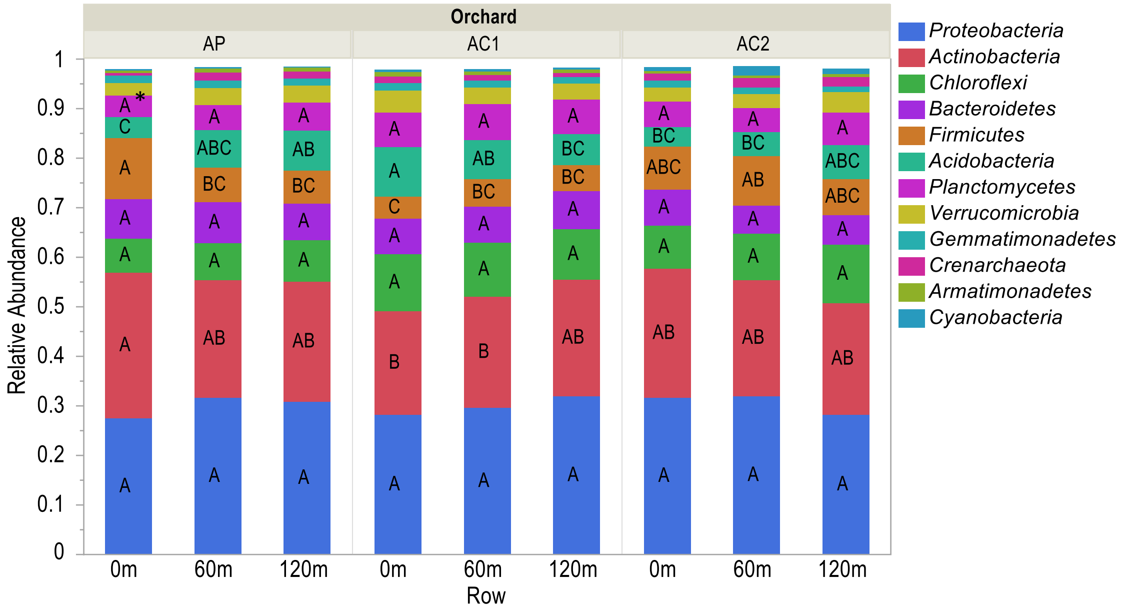

B. Families

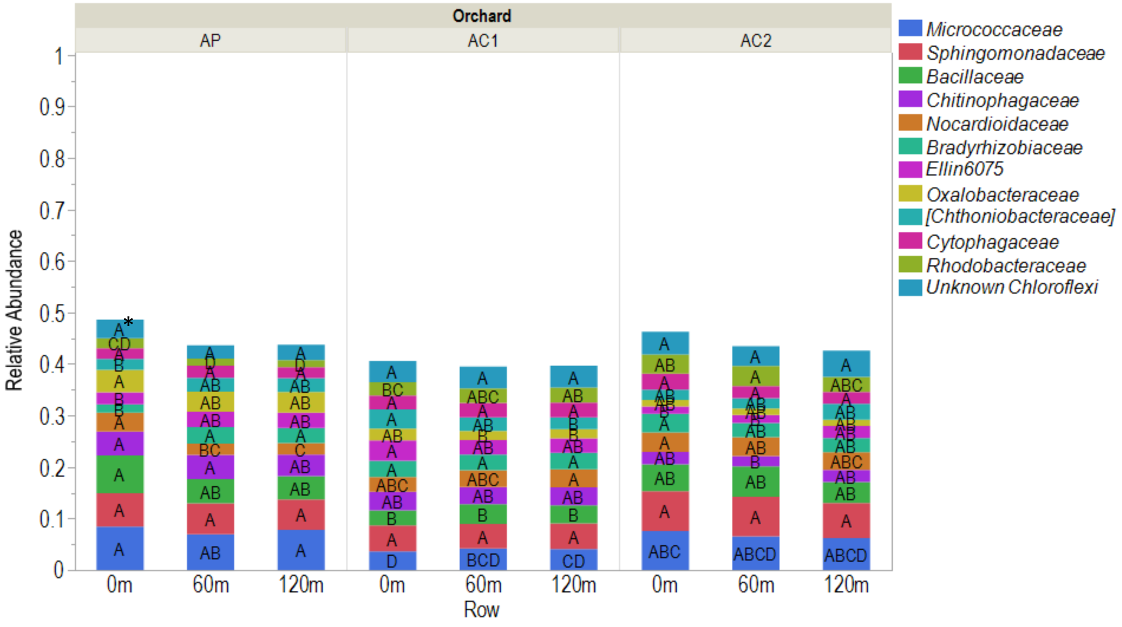

Figure S4. Relative abundance of the top 12 bacterial (A) phyla and (B) families in orchard soil samples. AP, *n* = 50; AC1, *n* = 45; AC2, *n* = 18.
*Within a phylum or family, different letters indicate significant differences in relative abundance among orchard rows. Statistical analysis is shown for the 12 families and the most dominant phyla

A. Phyla

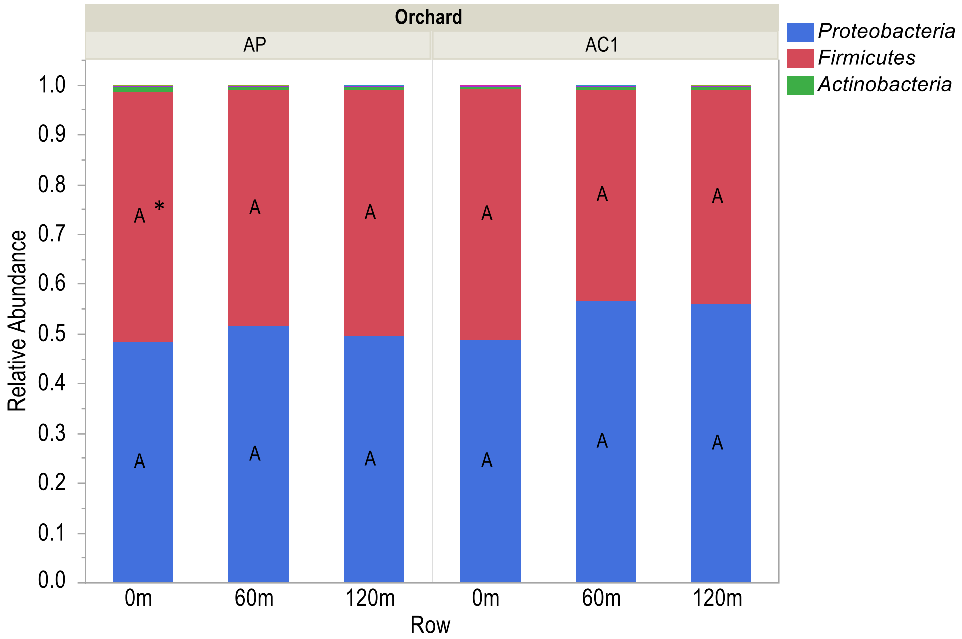

B. Families

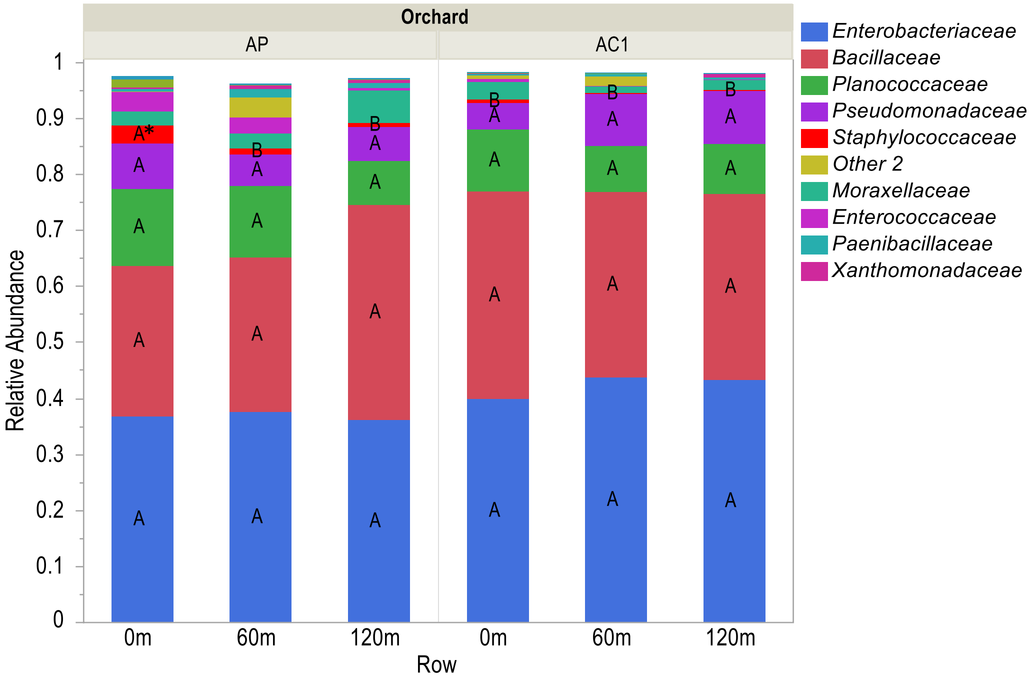

Figure S5. Relative abundance of the top 12 bacterial (A) phyla and (B) families in orchard air samples. AP, *n* = 23; AC1, *n* = 27.

*Within a phylum or family, different letters indicate significant differences in relative abundance among orchard rows. Statistical analysis is shown for the 12 families and the most dominant phyla.

A.

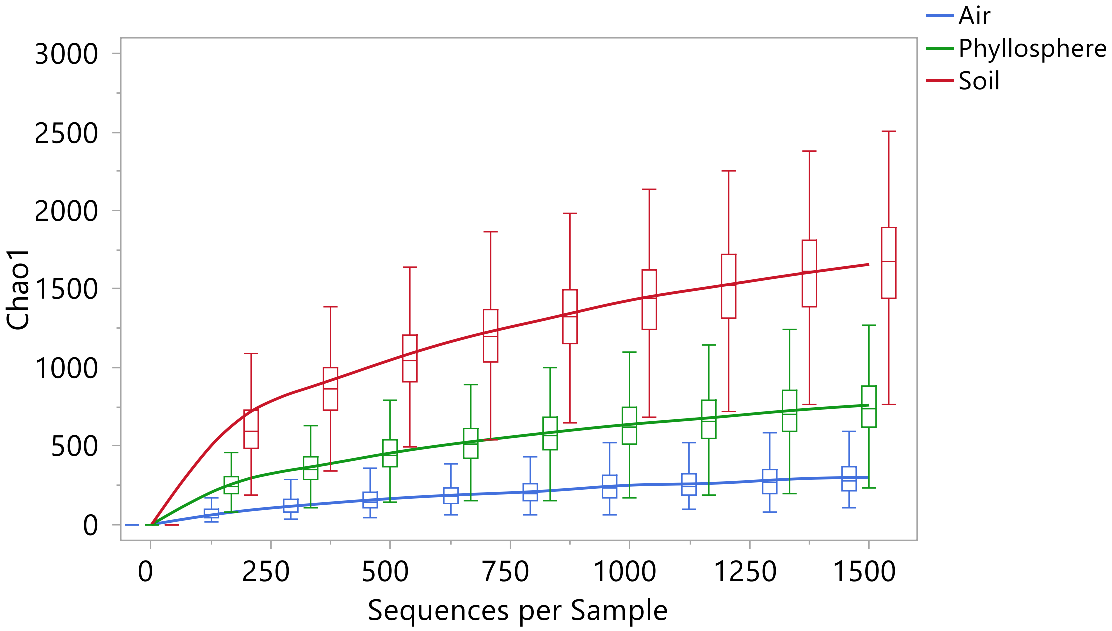

B.

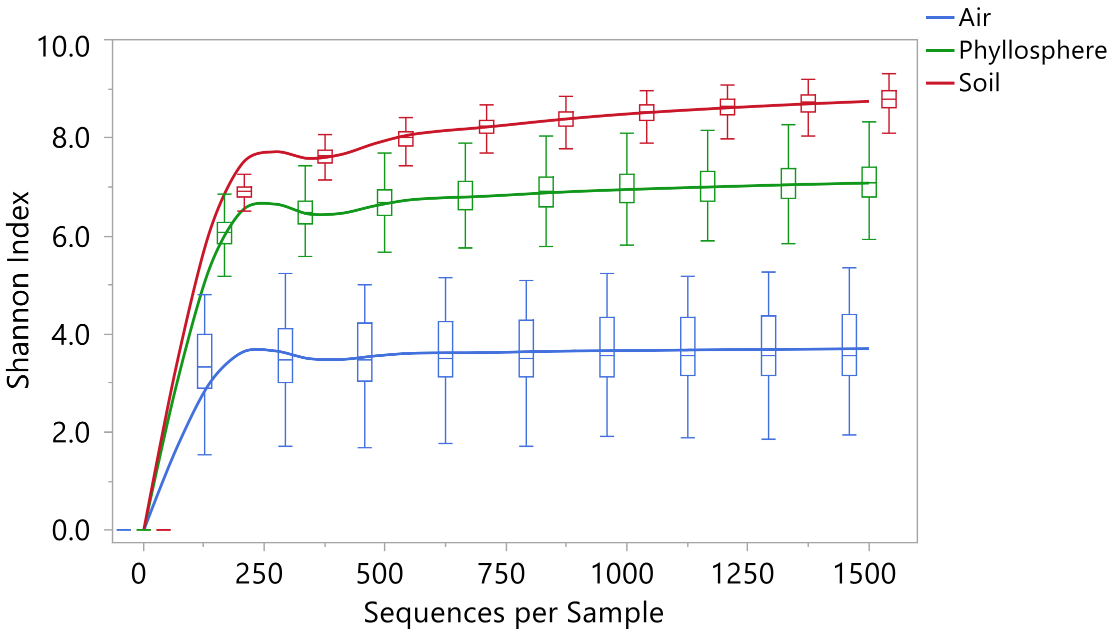

Figure S6. The alpha diversity metric Chao1 (A) and Shannon Index (B) by sequences per sample. Air, *n* = 50; Phyllosphere, *n* = 125; Soil, *n* = 113.
